# Hair Contamination of Sheepdog and Pet Dogs with *Toxocara cani*s Eggs

**Published:** 2012

**Authors:** M Tavassoli, S Javadi, R Firozi, F Rezaei, AR Khezri, M Hadian

**Affiliations:** 1 Department of Pathobiology, Faculty of Veterinary Medicine, Urmia University, Urmia, Iran; 2 Department of Clinical Science, Faculty of Veterinary Medicine, Urmia University, Urmia, Iran; 3 Private Clinician, Urmia, Iran; 4 Department of Pathobiology, Faculty of Veterinary Medicine, Razi University, Kermanshah, Iran; 5 Department of Natural Science, Hedmark University College, Hamar, Norway

**Keywords:** *Toxocara canis*, Dog, Public, Health, Zoonoses

## Abstract

**Background:**

We tried to investigate the hair contamination of pet dogs and farm sheepdog with *Toxocara* eggs in terms of the different sex and age groups in north-west of Iran (Urmia and its suburbs).

**Methods:**

Hair samples were collected from a total of 138 pet and farm sheepdogs from November 2008 to June 2009 in Urmia City and the suburb (West Azerbaijan-Iran) and examined for the presence of *T. canis* eggs.

**Results:**

*T. canis* eggs found in 60 samples altogether (pet and shepherd dogs) showed a contamination rate of 36.2%. The number of observed *T. canis* eggs in each microscope field was varied from 1 to > 400. The age of the dog was found a significant factor to influence the prevalence and intensity of contamination, with 82% of all the eggs recovered from puppies (six months and younger). Additionally, the numbers of eggs in farm sheepdogs were significantly higher than pet dogs (*P*<0.05).

**Conclusion:**

This report shows that direct contact with *T. canis* infected dogs, particularly puppies from shepherd dogs, may pose a serious hazard to human. Besides, as they may harbor a considerable number of eggs on their hair, they can contaminate the soil and the environment.

## Introduction

*Toxocara canis* as a cosmopolitan gastrointestinal parasite of canids, contaminate environment extensively by excretion of infective ova and put the exposing paratenic hosts, including humans, at the risk of infection (1). Infestation could be occurred by contact with contaminated soil, dirty hand and raw vegetable. Pica as a nutrition disorder has an important role in the infestation with *Toxocara* spp. (2). In human this parasite can cause significant clinical disease. For instance, the larvae of *T. canis* are capable of invading human tissues and causing Ocular Larva Migrans (OLM), Visceral Larvae Migrans (VLM), Eosinophilic Meningoencephalitis (EME) and/or Covert Toxocariasis (CT) (1).

Human infection with *T. canis* may result from the ingestion of an embryonated egg, and can produce a number of clinical syndromes and only a few larvae are needed to cause disease (3). Stray and farm sheepdogs in particular may represent a major source of *Toxocara* eggs due to the high number of *Toxocara* worms they harbor (4, 5). Dogs are infected by *T. canis* from various ways such as ingesting eggs, prenatal (transuterine) a clostral (lactogenic) transmission (6).

Epidemiology of human toxocariasis is complex and because the eggs require maturation period after ingestion from dogs the general consensus is that transmission to humans occurs mainly through contact with contaminated soils (7–9). However, the dog's hair contamination with *Toxocara* eggs is also thought to play an important role as stated by Wolfe and Wright, who proposed that humans may be infected through ingesting embryonated eggs which have been picked up directly from the coat of a dog. *Toxocara* spp. eggs possess a thick and complex shell layer enabling them to protect themselves from the influence of environmental factors (10). It seems as they can survive in the soil for a long time, they can also survive in animal hair/coat in favorable conditions to develop infective eggs (10).

The subject of *T. canis* eggs in dog's coats was first investigated by Wolfe and Wright (10). The fact that the soil contamination with the eggs in favorable conditions of highly populated area suggested was low strengthened the theory that eggs could embryonate on the coat of a dog and direct contact with dogs could be seen as an alternative explanation of the epidemiology of the disease (10).

Visceral Larvae Migrans is an endemic disease in Iran with a quite high prevalence rate especially among children (5.3%-25.6% in different parts of Iran) (11, 12). This merits more studies to be carried out regarding all aspect of the disease identifying potential public health risk factors.

The present study was designed to investigate the hair contamination of pet dogs and farm sheepdog with *Toxocara* eggs in terms of the different sex and age groups in north-west of Iran (Urmia and its suburbs).

## Materials and Methods

Urmia is the capital of the West Azerbaijan Province in north-west of Iran, with about 700000 inhabitants and a dog population of around 50000 dogs (13). This area is semi-humid, with mean rainfall of about 350 mm. The maximum mean monthly temperature of 28.3 °C in August and the minimum mean monthly temperature -5 °C in January.

Dog hairs from the different breeds of pet dogs attended the clinic of the Faculty of Veterinary Medicine, Urmia University, Iran, and farm sheepdogs from different farms were enrolled. The age and sex of each dog was recorded and an identification number allocated to each animal. Using tooth development scheme, the age groups were divided into 44 adults and 94 puppies (up to 6 months old). The breed, sex and age distribution of dogs are shown in Table 1.

**Table 1:** *Toxocara* eggs in the hair of 238 examined dogs

Breed	Age group (yr)	Sex	Positive samples	Negative samples
Farm sheepdogs	Puppy(<6m)		40	32
	Adult (6m <)		6	5
		Male	40	29
		Female	6	8
Pet Dogs	Puppy (<6m)		1	21
	Adult (6m <)		3	30
		Male	2	37
		Female	2	14

Collections of the samples were carried out from November 2008 to June 2009 in Urmia City and the suburb (West Azerbaijan-Iran). Hair was taken from the peri-anal region and dorsum of each dog and stored at 4 ^°^C until examined.

Hair samples from all dogs were clipped from the perianal region, the caudal aspect of the hindlimbs and the underside of the tail. The hair samples not stained with feces were weighed and their weight ranged from 0.1g to 0.6 g with the mean value of 0.4 g. The samples were stored at 4 ^°^C and examined within 2 weeks. Eggs were recovered from the hair using a modified method by Wolfe and Wright (10). Each hair sample was placed in 40 ml of water with 1 drop (approx. 75 ml) of Tween 80. This was vigorously shaken using a mechanical shaker for 2.5 min, the suspension was poured onto a 310 mm sieve, underneath which were a 210 and a 38 µm sieve. The hair was thoroughly washed over the sieves with copious amounts of tap water. The hair was removed from the top sieve and placed in 40 ml of water with Tween 80, shaken, and rewashed through the sieves. All the material trapped by the 38 mm sieve was collected using Pasteur pipettes and transferred to a centrifuge tube. The sample was centrifuged at 5000 rpm for 15 min. Finally the supernatant was decanted and the remaining plug put on a microscope slide and re-suspended in a drop of water. The sample was examined using a light microscope under ×40 magnification. Present an intensity of *T. canis* eggs were determined for each sample. Hair contamination in different sex, age groups, pet dogs and sheepdogs was analyzed using *x* ^2^ test (*P* ≤ 0.05). One way ANOVA was used to test differences in egg number of *T. canis* in different groups.

## Results

**Fig. 1:**
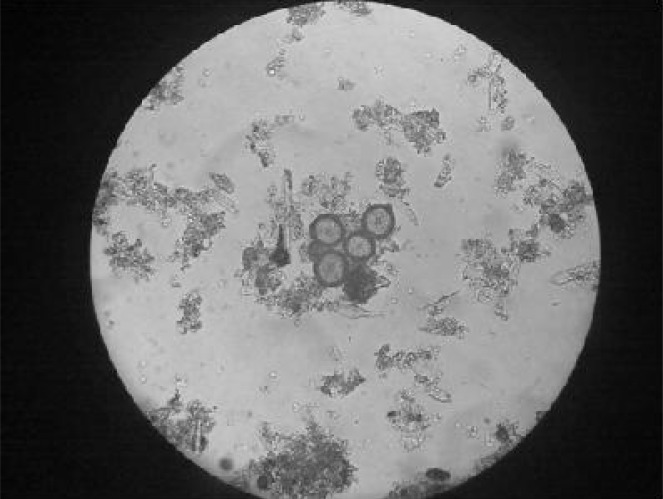
Ova of *Toxocara canis* in farm sheepdogs (x40) (Original)

The results indicated that *T. canis* eggs (Fig. 1) were found in 4 and 46 hair samples of the 55 and 83 pet and farm sheepdogs, respectively. Table 1 shows the prevalence of positive hair samples according to the age and sex class.

Over 82% of eggs all were recovered from the 94 puppies and only 18% of eggs were found on the 44 adult dogs (Table 1). About 39% of male dogs and 27% of female dogs were found positive for *T. canis* eggs in their hair. Puppies significantly had a higher egg number in their hair than older dogs (X_2_ = 6.959; *P* < 0.01). Moreover, egg numbers in the samples of farm sheepdogs were significantly higher than pet dog (X_2_ = 33.19; *P* < 0.01). However, study on egg numbers in different sexes of the dogs showed that there were no significant differences between male and female dogs (X_2_ = 1.518).

*T. canis* egg contamination in pet dogs was 7.3% (4 out of 55). The same value for farm sheepdogs stood at 55.4% (49 out of 83). Comparison of number of hair sample contaminated with *T. canis* eggs among farm sheepdogs and pet dogs revealed that there was no significant differences in positive samples between different age groups either in farm sheepdogs (P = 0.51, X_2_= 0.40), or in pet dogs (P = 0.51, X_2_ = 0.40). In addition, no significant differences were found between different sex groups and the hair contamination (farm sheepdogs: male = 69, female = 14; *P* = 0.32, X_2_ = 1.08 and pet dogs: male = 39, female = 16; *P* = 0.36, X_2_= 0.91).

## Discussion

Human risk to zoonotic parasitic infestation could be minimized by understanding of their epidemiology. Zoonoses involving dog parasites are both common and important, with some causing serious disease. In this regard, toxocariasis is an important zoonotic disease and a public health concern in most countries. Iran, as a developing country, with a quite high rate of toxocariasis among children is not an exception. The main risk factor in humans is the presence of dogs (in close contact with humans) parasitized by adult *Toxocara* worms, a situation that elicits soil contamination by *Toxocara* eggs. The contamination of dog's hair/coat with *Toxocara* egg and its importance has been the matter of interest in recent years. In 2003, Wolf and Wright seemed to be the first to undertake an investigation regarding *T. canis* eggs in dogs’ coats (10). Roddie et al. put the dogs in three classes (puppy, juvenile and adult) and found that puppies significantly had higher egg numbers in their coat than others (3). *T. canis* eggs were found in 21.56% of dogs’ coats (14). In the current study, 36.2% of hair samples of examined dogs were contaminated with *T. canis* eggs. Similarly, 25% of examined dogs had *T. canis* eggs in their coats (10).

The prevalence of soil contamination by *Toxocara* spp. eggs in various regions of world varies from 1.2% to 92% (15–22, 13). The mean rate of *Toxocara* spp. eggs was found 0.09 per 30g in soil samples from all parks studied and 0.6 per 30g in soil samples from the contaminated parks (6). The number of eggs in positive samples of examined soils varied from 2 to 22 (per 100g) (23). 7.8% of samples collected from public parks contaminated with *Toxocara* spp. eggs (13). Eggs in each microscopic field were varied from 1-8. There was no evidence of a direct link between seroprevalence in people and soil contamination (10), however, Mizgajska claimed to provide the evidence for such an association (19). 9.7% of pet dog feces were positive for eggs of *Toxocara* spp. in Urmia region (24). Some studies from various countries showed that the seroprevalence of *Toxocara* infection was independent to soil contamination by *Toxocara* spp. eggs. For example, in Anse-la-Raye, St. Lucia, seroprevalence of toxocariasis in human was found 86% among children (25), while soil contamination was 6.6% (26). In addition, in Nigeria, soil samples contamination with *Toxocara* spp. eggs were 13% (16), whereas toxocariasis seroprevalence was 29.8% in same country (27). It seems that the soil contamination levels alone cannot account for some of high seroprevalence thus indicating that direct contact with dogs might be responsible for such high seroprevalence levels.

The prevalence and intensities of different parasites infections were significantly higher in local breeds and their crosses than in exotic breeds (28). The prevalence of most parasites was similar for dogs of mixed-breed and for dogs of a defined-breed except for *Cystoisospora* spp. and *T. canis*, which showed a significantly higher prevalence in mixed-breed dogs (29). In the current study, farm sheepdogs showed a significantly higher number of *T. canis* eggs in their coats compared with pet dogs. But this seems mostly due to a life-style and absence of prophylactic treatment programs in farm sheepdogs than their breed or type of coat in these dogs. However, the type of coat may provide a suitable environment, namely moist for the development of *T. canis* eggs (14).

Puppies and adults may also differ in the source of the eggs found on their hair. Roddie et al. listed a number of reasons for suggesting this (3). The high *T. canis* prevalence in puppies is associated with the life cycle of the parasite which involves prenatal and transclostral transmission, while resistance develops to the parasite in older dogs (30). We found that puppies in both farm sheepdogs and pet dogs significantly had higher *T. canis* eggs contamination than adult dogs. This data agreed with the results of others (28, 29, 31, 32). In the current study, no significant differences was found between male and female dogs in both groups, showing that both sexes seemed to have similar resistance to *T. canis* infections, whereas it has been reported that female dogs near to their parturition may have more inclination to develop of dormant larva of *T. canis* (30).

## Conclusion

The results of this study highlight the importance of regular anthelmintic medication particularly in puppies and farm sheepdogs in order to minimize the risk of transmission of *T. canis* eggs to humans. In addition, anthelmintic therapy in dogs could help to control others important parasitic diseases such as echinococcosis in these animals which are equally of public health importance.
